# First Insights into the Population Genetic Structure and Heterozygosity–Fitness Relationship in Roe Deer Inhabiting the Area between the Alps and Dinaric Mountains

**DOI:** 10.3390/ani10122276

**Published:** 2020-12-02

**Authors:** Elena Buzan, Urška Gerič, Sandra Potušek, Katarina Flajšman, Boštjan Pokorny

**Affiliations:** 1 Faculty of Mathematics, Natural Sciences and Information Technologies, University of Primorska, Glagoljaška 8, 6000 Koper, Slovenia; elena.buzan@upr.si (E.B.); urska.geric@famnit.upr.si (U.G.); sandra.potusek@famnit.upr.si (S.P.); 2 Environmental Protection College, Trg mladosti 7, 3320 Velenje, Slovenia; 3 Slovenian Forestry Institute, Večna pot 2, 1000 Ljubljana, Slovenia; katarina.flajsman@gozdis.si

**Keywords:** *Capreolus capreolus*, microsatellites, population structure, heterozygosity, fragmentation, body mass, reproductive potential

## Abstract

**Simple Summary:**

We determined the genetic variability, population structure, and influence of genetic factors on two parameters of fitness (body mass and reproductive ability) in roe deer females in the contact zone between the Alps and the Dinaric Mountains by utilizing microsatellite variations in 214 individuals collected throughout Slovenia, Central Europe. Spatial differences in the genetic diversity of the species can be explained by population history, different approaches to population management and/or different connectivity among subpopulations. The population genetic structure confirms the high side fidelity of roe deer, but also shows the existence of admixtures of genes among different areas. We found evidence that genetic factors, including individual heterozygosity, influence body mass, confirming that heterozygosity positively affects fitness in wild populations. However, as the effect of genetic factors is usually masked or overruled by the influence of environmental factors, i.e., availability of resources, data on the joint influence of external and intrinsic factors on fitness and other life-history traits are needed to better predict the population dynamics of targeted species, which would enable sustainable, science-based population management.

**Abstract:**

Across its pan-European distribution, the European roe deer (*Capreolus capreolus*) faces a wide diversity of environmental and climatic conditions; therefore, several factors, including intrinsic ones, shape life-history traits and cause significant variability in parameters of fitness. By utilizing microsatellite variations in 214 roe deer females collected throughout Slovenia, Central Europe, we determined the genetic variability and population structure of this species in the contact zone between the Alps and the Dinaric Mountains, i.e., over a wider area where data on the genetic outlook for this—the most common and widespread European wild ungulate—have been completely lacking so far. Throughout the country, we found moderate microsatellite diversity (Ho = 0.57–0.65) in relation to the observed heterozygosity reported for other roe deer populations in Europe. Spatial differences in genetic diversity of the species in Slovenia can be explained by population history linked to varying approaches to population management and/or different connectivity among subpopulations in topographically differentiated habitats. A country-wide pattern of genetic structure is clearly defined by separation of the populations into three groups present in the following regions: (i) Southern sub-Mediterranean and Karst regions, (ii) Central Slovenia, and (iii) the Sub-Pannonian Region in the north-east. This is also confirmed by evidencing a moderate isolation by distance, especially by separating southern samples (coastal Slovenia) from others. Levels of genetic differentiation vary among populations, which can be explained by the effect of natural geographical barriers or the presence of anthropogenic barriers such as urban areas and highways. In the subset of 172 yearling females, we analyzed the influence of genetic advantage (individual heterozygosity) and other genetic data (reflected in the structuring of the population) on body mass and reproductive ability. We found evidence that genetic factors influence the body mass of roe deer yearling females (explaining altogether 18.8% of body mass variance), and the level of individual heterozygosity alone also positively affected body mass, which is in accordance with the theory that heterozygosity is commonly positively correlated with fitness in wild populations. However, we did not uncover any effect of heterozygosity on two parameters of reproductive ability (fertility and potential reproductive outcome), indicating that several other factors, especially environmental ones, have a predominant effect on the parameters of fitness in roe deer.

## 1. Introduction

European roe deer (*Capreolus capreolus*) is spread throughout Europe from the Mediterranean to Scandinavia [1]. Across its wide distribution range, the species faces a huge diversity of environmental and climatic conditions. Therefore, several factors shape its life-history traits and cause significant variability in its genetic structure and parameters of fitness [2]. This variability is also high due to anthropogenic and historical reasons. The climatic changes during the Pleistocene led to repeated range shifts of roe deer (for review, see [3]) and major genetic divisions within this widespread species. Indeed, several studies, mainly based on mitochondrial DNA (mtDNA) information (e.g., [4,5,6,7,8,9]), revealed that roe deer populations in Europe express significant structuring consistent with cycles of restriction and colonization during glacial–interglacial periods. Vernesi et al. [7] and Randi et al. [8] identified three main mtDNA lineages of roe deer in Europe, which were named according to their geographical distribution: Western, Central, and Eastern.

In Slovenia, the country where we conducted this study, the results of Plis et al. [10] showed the existence of the Eastern and Central genetic lineages of roe deer with a broad contact zone spreading across the country. Here, the history of the roe deer which predominantly affects the genetic outlook of the species was very dynamic. Until the mid-19th century, roe deer had mainly occupied large deciduous forests in the contact zone between the Alps and the North-west Dinaric Mountains (currently the central part of Slovenia). Outside this area, only a few small colonies existed in the south and north. At the beginning of the 20th century and primarily after World War II, improved habitat conditions enabled fast population growth of the central population. This resulted in expansion of the species southwards into the sub-Mediterranean and Karst regions, into hilly areas in the north and north-east, and finally in the appearance of large lowland ecotype groups in the open agricultural lands of the sub-Pannonian Region [11,12]. The population reached its peak in the mid-1990s [13]. Today, in spite of a local decrease, roe deer are widely distributed throughout the country [14] with a population size estimated at >200,000 individuals [15].

The species exhibits a similar history on the continental scale: An evident increase both in abundance and geographic distribution throughout Europe in the 20th century made roe deer a prime example of a successful species [16]. However, since the mid-1990s, European roe deer in many areas have experienced considerable reductions in population numbers due to habitat degradation, the spread of agriculture, too intensive selective hunting, and other human-induced disturbances, which have significantly affected the size, structure, and dynamics of populations [17,18]. Populations that were affected either historically or in more recent times by strong declines may recover quickly, but a long-term effect on a genetic diversity (both through the initial loss of allelic diversity and through genetic drift over time) may remain.

As a species which is widespread throughout the Europe [1], roe deer can serve as a model species for studying the genetic effects of habitat fragmentation and restoration, as well as of management and human disturbances. For example, it has been shown that the combination of several landscape features, particularly barriers (i.e., motorways, rivers, canals, heavily populated urban areas) can lead to population genetic differentiation [19,20,21], and that genetic discontinuities correlate with the presence of transport infrastructure [22,23]. Moreover, roe deer is a suitable model species for studying factors shaping the population’s genetic outlook as it is pronouncedly philopatric, i.e., individuals of both sexes maintain small home ranges (usually <100–150 ha) for many years [24,25]. In addition, the ecological features and social behaviour of roe deer are fairly well understood [1,24,26,27,28,29], which may help in working out the effects of intrinsic (genetic) factors on its life-history traits and fitness.

As roe deer are income breeders [29], their body mass shows a relatively low seasonality due to investment in reproduction; indeed, during the rut, roe deer males lose on average only 7.5% of body mass compared to 19.5% in red deer (*Cervus elaphus*) and 16.0% in chamois (*Rupicapra rupicapra*) [30]. Therefore, roe deer body mass can serve as a proxy for an individual’s general fitness, and is of crucial importance for the reproductive success of the species (reviewed in [31,32]). Indeed, the body mass of female roe deer has a positive effect on their reproductive ability, i.e., on the probability of ovulation and the potential litter size (reproductive outcome), which is particularly pronounced in primiparous does [33]. Body mass and reproductive potential of the individuals and their average values in a given population are affected both by environmental factors and the individual characteristics of the animals. A meta-analysis of all published European studies on roe deer reproduction showed that the body mass of roe deer females as well as their litter size increase with latitude [32]. At the same latitude, body mass increases with landscape openness, probably due to the availability of nutrient-rich foods provided by meadows and cultivated crops [34]. In Slovenia, female roe deer inhabiting the open agricultural areas of the sub-Pannonian Region have on average significantly higher body mass and reproductive potential compared to forest roe deer from other areas, while roe deer from the sub-Mediterranean area are lighter and produce fewer offspring [35].

Assuming that body size (and consequently body mass) is hereditary, a phenotypic response to selection in the form of an increase in body size could be expected, but this fact is rarely observed in nature. While there is a general consensus that selection is stronger under harsh conditions [36], it remains unclear whether genetic variability should increase or decrease the selection under increasingly stressful conditions in changing environments [37]. Indeed, in contrast to the effects of different environmental factors, age, and phenotypic characteristics of individuals on their fitness, the influence of genetic variability/vigour (e.g., heterozygosity) on the main fitness parameters of roe deer, such as body mass and reproductive ability, has been rarely and only indirectly studied so far (see [38,39]). To fill this gap in our knowledge, we analyzed neutral genetic variation and its possible influence on body mass and reproductive capacity (fertility, potential reproductive outcome) in a large sample set of yearling females collected over the entire gradient of species occurrence in Slovenia. We used microsatellites to analyze the genetic variation and structure of roe deer from different geographical areas with profoundly different histories to estimate the sub-structuring of the population, discussed possible explanations for the determined genetic structure and its geographic differentiation, and tried to determine whether genetic variability (multilocus heterozygosity) and/or genetic clustering (reflecting spatial genetic population structure) of individual roe deer females have any important effect on their fitness (measured by body mass and reproductive potential).

We tested the following hypotheses: (i) The genetic structure of Slovene roe deer population is determined by landscape features and is an expression of isolation by distance; and (ii) mean microsatellite heterozygosity is positively correlated with individual body mass and has an effect on the reproductive capacity of primiparous roe deer females, both suggesting that genetic features have an important influence on roe deer fitness and the population increment rate.

## 2. Material and Methods

### 2.1. Ethical Statement

All harvested individuals used in the study were legally hunted during the regular hunting activity prescribed by the state of Slovenia within yearly hunting management plans. We only used data on dead individuals and no animal was either shot or killed by any other means for the purposes of this research. Therefore, there was no need to seek ethical approval.

### 2.2. Study Area and Sampling

**Figure 1:**
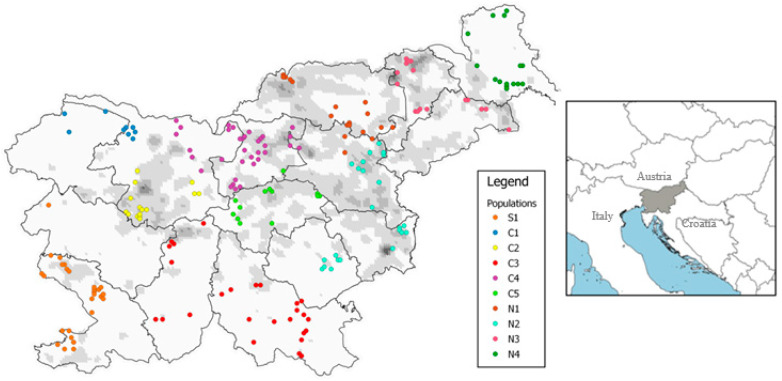
Sampling sites of roe deer females included in the study. Different colors indicate different a priori recognized “populations” (see Table 1 for geographic names of the studied areas, and Table S1 in Supplementary Materials for attributive data on individuals). The borderlines separate the 15 hunting management districts, while the grey background indicates the gradient in population density of roe deer in Slovenia (white: 0–9 individuals/100 ha; black: 40–49 individuals/100 ha; after [14]). The insert shows the location of the studied area (whole Slovenia) in Europe.

In this study, 214 roe deer females (172 yearlings) from all 15 Slovene hunting management districts (HMD) were used; on the basis of the location of mortality, the studied individuals were pooled prior to analysis into 10 groups (for the purposes of this paper further considered as “populations”) based on the geographical characteristics of Slovenia (Figure 1, Table 1; for details on each individual, see Supplementary Material, Table S1). Borders among groups were not necessarily consistent with borders among HMD; rather, we a priori assessed that studied groups could be (partially) isolated from each other considering the landscape features. Moreover, we also considered that these geographical areas had experienced different historical management of roe deer. In the 19th century, the central area (populations C1–C5) was part of the historical region of Carniola under the Austro-Hungarians and had well-established management and regulated annual culls. By contrast, during the same period, populations in the south-western region (S1) and in the northern and north-eastern regions (N1–N4) were rare and almost extinct due to unsustainable hunting and poaching [11,12]. However, nowadays roe deer is abundant throughout Slovenia and is the most common and important game species in the country.

Muscle tissue or a biopsy of the reproductive organs of female roe deer, shot or found as roadkill during the hunting season (1 September–31 December), were used. All samples were collected from 2013–2015 in 58 hunting grounds, continuously distributed throughout Slovenia, and covering the whole spectrum of environmental conditions and population characteristics of the species’ distribution in the country. Slovenia (20,271 km^2^) is located in Central Europe (46° N, 14° E) at the intersection of four major European geographical units: The Alps, the Pannonian Basin, the Dinaric Alps, and the Mediterranean. The climate is varied with a continental climate in the north-east, a severe alpine climate in the high mountain regions, and a sub-Mediterranean climate in the coastal region.

We included only females (primarily yearlings) in the study due to the following facts: (i) Roe deer females show high spatial fidelity [40] and have considerably smaller home ranges than males [25]; therefore, we minimized the influence of spatial behaviour, i.e., dispersions, seasonal migrations, and roaming, on the spatial genetic structure revealed by our analyses; (ii) a recent genetic study of paternity and relatedness of roe deer in Slovenia revealed that in yearlings, females were shot much closer to their mothers (and thus presumably close to their natal location) than males [41], confirming that employment of this demographic category would minimize the risk of disturbing effects of spatial behaviour, i.e., long-distance movements outside the natal home range; (iii) in roe deer females, yearlings have much higher variability in reproductive potential compared to adults in which variation in both ovulation probability and litter size is very low [33]; therefore, any effects of genetic features on reproductive ability is expected to be seen primarily in yearlings.

Since all studied females were shot during regular hunting or were found as road-kill in the autumn, when roe deer females are in embryonic diapause [42], we determined the reproductive potential of each doe by counting the number of corpora lutea in the ovaries (representing the ovulation rate). By doing this, we measured the reproductive capacity in the early stage of reproduction, i.e., before possible implantation losses, which can be affected by different individual and environmental factors in roe deer [43]. Thus, in terms of detecting the possible effects of genetic features on the reproductive potential of roe deer females, the ovulation rate is a much more reliable trait than the number of fetuses would be in later, more costly, stages of reproduction.

### 2.3. Data Collection and Preparation

Immediately after the culling and dissection, hunters placed the uterus (or muscular tissue in some cases) in plastic bags and kept them frozen until collection. For each specimen, the date and place of sampling, the eviscerated carcass mass (total body mass less viscera and flowing blood, but with head and feet on; measured on ±0.5 kg precision), and the age group (yearling, adult) were recorded immediately after the hunting episode (see Table S1 in Supplementary Material). The lower jaw was also removed and the left hemimandible was kept for the age assessment confirmation, which was carried out in parallel by two co-authors (K.F. and B.P.) by macroscopic inspection of teeth development and tooth-wear (described in [44,45]).

Since the body mass of yearling females in our sample set linearly increased with consecutive days in the September–December period, the eviscerated body mass of each individual was subsequently corrected (standardized) on a daily basis using a general regression model (for details, see [33]). Standardized body masses were used in all analyses and are presented in the paper wherever body mass is referred to (in spatial context, they are visualized in Supplementary Material, Figure S1).

To determine the fertility and ovulation rate (potential litter size) of each female, the presence and number of corpora lutea (CL) had been previously determined by ovarian dissection [33,35] (see Supplementary Material, Table S1 and Figure S2). Parallel to the analysis of fertility, 1–3 g of the uterus of each female were stored in ethanol for subsequent DNA analysis.

### 2.4. DNA Extraction and Microsatellite Genotyping

Tissue samples (2 × 2 mm) were air-dried under sterile conditions to remove the ethanol and the DNA was extracted using a peqGOLD Tissue DNA Kit (S-Line) (VWR International, Leuven, Belgium) in accordance with the manufacturer’s instructions. The DNA was eluted in the appropriate kit elution buffer, then the sample concentrations were normalized and the dilutions stored in the refrigerator at 4 °C to shorten the thaw–frost cycles. The concentration and purity of the DNA obtained in the final elution volume were measured with a 3.0 Qubit Fluorimeter (Life Technologies, Carlsbad, CA, USA) using Qubit^®^ dsDNA (Invitrogen BR Assay Kit, Carlsbad, CA, USA). In addition, the spectral curve was measured on Epoch™ (BioTeck Microplate Spectrophotometer, Winooski, VT, USA) using Gene5 v.1 software to check for potential impurities. We expressed the amount of DNA obtained in ng per µL of DNA in the final elution volume.

The microsatellites (Supplementary Material, Table S2 [46,47,48,49]) were amplified with the ready-to-use KAPA2G Fast Multiplex Mix (Kapa Biosystems, Wilmington, MA, USA), in accordance with the manufacturer’s instructions, using 3 µL of template DNA and 0.3 mM of final concentration for each primer used in the set. Amplification was performed under the following conditions: Initial PCR activation for 3 min at 95 °C, followed by 35 cycles of denaturation for 15 s at 95 °C, annealing for 30 s at 58 °C, extension for 30 s at 72 °C, and a final extension for 10 min at 72 °C. Fragment analysis was performed on a SeqStudio sequencer (Thermofischer scientific, San Jose, CA, USA) using the GeneScan LIZ500 (-250) standard (Applied Biosystems). The results were validated with GeneMapper v.5.0 software (Applied Biosystems). We amplified 14 microsatellite loci in 4 multiplex sets containing 4, 2, 3, and 4 microsatellites. The locus MAF70 was amplified separately using the protocol described by [22], which is commonly used in population genetic studies using DNA from deer muscle tissue (for details, see [50]).

The deviation from the Hardy–Weinberg equilibrium (HWE) was calculated with the Genepop 4.2 software [51]. The exact test to assess heterozygosity deficiency was performed for each population occupying different habitat patches. The baseline significance level was set at 0.05 and a Bonferroni procedure was applied in multiple comparisons to compensate for the risk of a bloated type 1 deficiency. The presence of zero alleles can cause a significant heterozygote deficit and deviation from HWE. Therefore, using the FreeNA program [52], we estimated the proportion of null allele (NA) at each locus in each population.

The mean number of alleles (A), observed (H_O_) and expected (H_E_) heterozygosity [53], and inbreeding coefficients (F_IS_) were calculated for each population with Genetix 4.05.2 [54]. The allelic richness (AR) in populations was estimated using the rarefaction method in the program FSTAT 2.9.4 [55].

### 2.5. Determination of Genetic Variability among Populations

The FreeNA program was used to estimate the global F_ST_, with 1000 permutations. Estimates of pairwise F_ST_ were implemented in Genepop according to Weir and Cockerham (1984), and significant differences from zero F_ST_ estimates were tested again with 1000 permutations [56].

We performed discriminant analysis of principal components (DAPC), a method that allowed us to describe genetic diversity and identify genetic clusters using multi-variant methods. DAPC specifically seeks synthetic variables (the discriminant functions) that best reveal differences among groups while minimizing variation within clusters [57]. Furthermore, by performing the analysis on synthetic variables, we were able to speed up the clustering algorithm without losing information. To first identify the optimal number of clusters (K) that roe deer could be divided into, we ran successive k-means with an increasing number of clusters (K) using the function find.clusters from the R-package adegenet [57]. For each model, a statistical measure of goodness of fit (i.e., the lowest Bayesian information criterion) was computed, which allowed us to select the optimal K-value. We then performed DAPC using the dapc-function from the R-package adegenet to describe diversity between pre-defined clusters.

Software Structure 2.3.4 (Department of Statistics, University of Oxford, Oxford, UK) [58] and Geneland version 4.9.2 (Centre de Biologie et de Gestion des Populations, INRA-ENSAM-CIRAD-IRD Montpellier, France ) [59] were used to analyze the population structure. The program Structure classifies individuals into a set number of clusters (K) so that the HWE in these clusters is achieved. In the Structure, ten independent runs were performed for each value of K ranging from one to ten using a model assuming correlated allele frequencies. Each run comprised a burn-in period of 100,000 replications followed by a run length of 100,000 Markov Chain Monte Carlo (MCMC) iterations. The results of the replicated runs for each value of K from two to ten were combined using Structure Harvester web v0.6.94 [60], and the optimal value of K was selected using the ΔK method developed by Evanno et al. [61]. Twenty independent runs were performed, setting K to the estimated optimal number of clusters using a burn-in of 100,000 and 1,000,000 MCMC iterations. The results of replicated runs were combined using the Greedy algorithm of Clumpp 1.1.1 [62], and the summary outputs were displayed graphically using Distruct 1.1 [63].

Several methods of Bayesian clustering are available that implement the spatial analysis of genetic data [64]. We performed several runs in Geneland to adjust the values of the input parameters based on the behaviour of the MCMC. This method ensures that the maximum parameter values were large enough to allow the MCMC to examine all likely regions of the parameter space, and confirm the convergence of the chains at the end of the runs. We then performed ten runs, consisting of a burn-in period of 50,000 MCMCs followed by 500,000 iterations, each with the selected parameter set for which K could be varied. We assumed that yearlings were sampled in or very close to their natal areas. Therefore, the uncertainty associated with the spatial coordinates was set at 1000 m. Two allele frequency models are available at Geneland. We used the model with correlated allele frequencies among populations. K was derived because the modal number of genetic groups was estimated as the best among 500,000 iterations of the ten runs. To select the best run, we used the posterior density of the runs as an estimate of their quality: The posterior density was estimated for each parameter set studied along the Markov chain, and represents the posterior probability of this parameter set.

We then investigated roe deer genetic structure with the R-package, adegenet 2.0.0 [65], using RStudio and R version 3.6.2 [66]. This package uses a multivariate method, i.e., discriminant analysis of principal components (DAPC; [67]), to identify the most likely number of clusters (K) or subdivided groups of genetically similar individuals.

The analysis of molecular variance (AMOVA; [68]) implemented in the poppr package, v2.8.2 [69] was performed in the R-package to test the genetic differences among individuals and populations, as well as between the optimal number of clusters identified by the Structure (K = 3). Statistical significance of the variance components was investigated using 999 permutations in ade4, v1.7-13 [70]. Finally, we tested for the presence of an isolation-by-distance (IBD) pattern between all populations [71,72] using the adegenet package in the R. Using a Mantel test (999 permutations; [73]), we tested the correlation between Edwards’ distances and Euclidean geographic distances [74,75].

In all statistical analyses, *p* < 0.05 was set as the level of statistical significance; in the case of stronger connections, relevant *p*-levels are explicitly indicated.

### 2.6. Effects of Genetic Features (Heterozygosity) on Body Mass and Reproductive Ability

A sample subset of 172 yearlings (i.e., in the age between 16 and 20 months) were used in this analysis. By using linear models, we analyzed effects of genetic features (individual multi-locus heterozygosity (HL), and a proportion of the membership of the individual in each of the three K-groups (q-values) estimated by the Structure) on standardized body mass as dependent variable.

We calculated HL as a measure of individual heterozygosity at multiple loci [76], using the R function genehet [77]. HL is a complex estimator that gives weight to more informative loci (e.g., loci with more alleles that are more evenly distributed). In simulated populations subjected to migration and admixture, HL better correlates with the inbreeding coefficient and with genome-wide heterozygosity than other heterozygosity indices, thereby reducing the sample size required to detect a heterozygosity–fitness correlation due to inbreeding [76].

Spatial algorithms in the program Structure assume a priori that all individuals are equally likely to be assigned to a genetic cluster, regardless of their geographical location or biological feasibility or constraint. Firstly, the Bayesian iterative algorithm randomly assigns individuals to a pre-determined number of groups, then variant frequencies are estimated in each group and individuals re-assigned to genetic groups/clusters (K) based on those frequency estimates (subset of allele frequencies identified in the data). The individual Q-matrix presents the inferred ancestry components of each individual in each of the K clusters (ancestry membership proportions of each individual). We included the membership proportion (q-values) of an individual’s assignment to the most probable number of clusters (K = 3) in the linear regression model (LRM) to better represent the overall pattern of genetic variability after testing the q-values for collinearity with HL by using an accepted threshold of −0.7 in the Pearson correlation [78].

By using the general linear model (GLM), we analyzed the effects of genetic features on fertility (presence/absence of CL) and potential reproductive output (number of CL in fertile individuals) separately, as these parameters may respond differently to various influential factors [79]. As the effect of body mass on both parameters of the reproductive potential of yearling females included in our study was already confirmed by Flajšman et al. [33], we aimed to explore an additional cofactor, i.e., contribution of individual heterozygosity to fertility (yearlings with vs. without CL; binomial distribution), and to the potential litter size of fertile individuals (number of CL = 1, 2; binomial distribution) as dependent variables. In GLM for fertility, in addition to genetic features (HL, q-values) the year of sampling was also included as a fixed factor (see [33]). We built all possible models and used the Akaike information criteria (AIC) to select the best and other still informative models with ΔAIC <2. These statistical analyses were performed using the lme4 package [80].

## 3. Result

### 3.1. Intra-Population Genetic Diversity

A total of 14 loci were examined in 214 individuals from 10 geographic regions (Table 1; Supplementary Material, Table S1). All loci were polymorphic, with the exception of locus NVHRT73. Two loci (ETH225 and NVHRT24) showed a deficit of heterozygotes, with significant results in 9 out of 10 populations. These two loci also had the highest overall estimates of null alleles frequency (NAF): ETH225 = 0.220, NVHRT24 = 0.058, and were therefore also excluded from follow-up analyses (Supplementary Material, Table S3). In the remaining 11 microsatellite loci (with less than 5% of NA across all populations), the average NAF per locus ranged from 0.005 (MCM64) to 0.045 (Roe8) with an average of 0.022. Seven out of 110 comparisons of loci by sample location deviated significantly from Hardy–Weinberg expectations.

Populations C2 (Polhograjsko and Škofjeloško hills), C3 (Dinaric Mountains), and N4 (Prekmurje) showed significant deviations from HWE based on F_IS_ (significantly positive values) (Table 1) but not based on exact tests in Genepop. The number of alleles per locus (A) ranged from 2 to 11 with a mean of 7.3. AR across populations ranged from 4.45 to 5.01, with the highest value in Kamniško-Savinjske Alps (C4), and the lowest in Julian Alps (C1) and Dinaric Mountains. A similar pattern was also observed for Ho with values between 0.58 and 0.65, and He with values between 0.61 and 0.66, where C3 had the lowest Ho.

### 3.2. Spatial Genetic Structure

The global F_ST_ value for ten populations was 0.017 (95% CI: 0.011–0.022) and differed significantly from zero. The pairwise F_ST_ values between populations (range: 0.006–0.040; mean ± SD: 0.017 ± 0.015) were also significantly different from zero (Table 2). The highest F_ST_ value was observed between population S1 (Coastal Slovenia) vs. C1 and C2, respectively.

**Figure 2:**
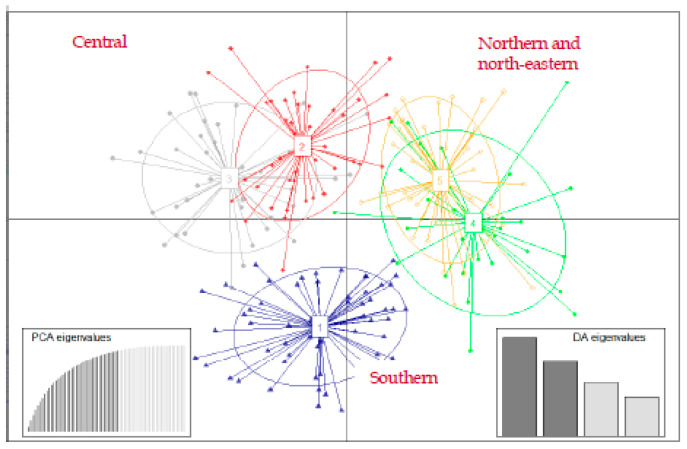
Discriminant analysis of principal components (DAPC) plots of genetic clusters of roe deer in Slovenia. The axes represent the first two linear discriminants (LD). Each ellipse represents a cluster and each dot represents an individual. Numbers represent the different clusters identified by DAPC analysis.

DAPC from the Bayesian Information Criterion suggested that there were five genetic clusters, clearly distinguishing individuals by their geographical origin (north–south gradient) and closest populations (Figure 2). The first principal component (PC) differentiated four clusters from the fifth, and the second PC displayed a slight differentiation among all five clusters. However, except for the cluster 1, the ellipses delineating the spatial extent of clusters were substantially overlapping, suggesting weak genetic structuring between them.

**Figure 3:**
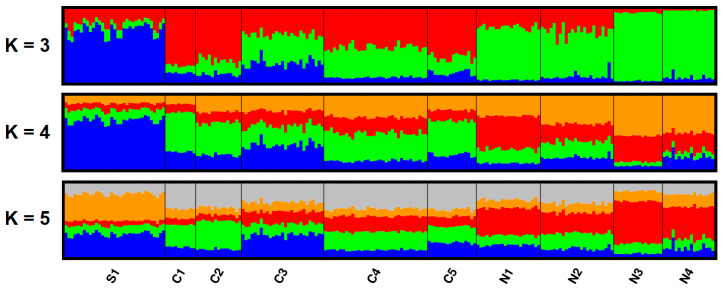
Genetic structure (from the Structure) of Slovene roe deer from 10 sampling areas/populations (see Table 1). Each individual is represented by a line proportionally partitioned into colour segments corresponding to its membership in particular clusters. Black lines separate individuals from different areas. K: number of clusters.

**Figure 4:**
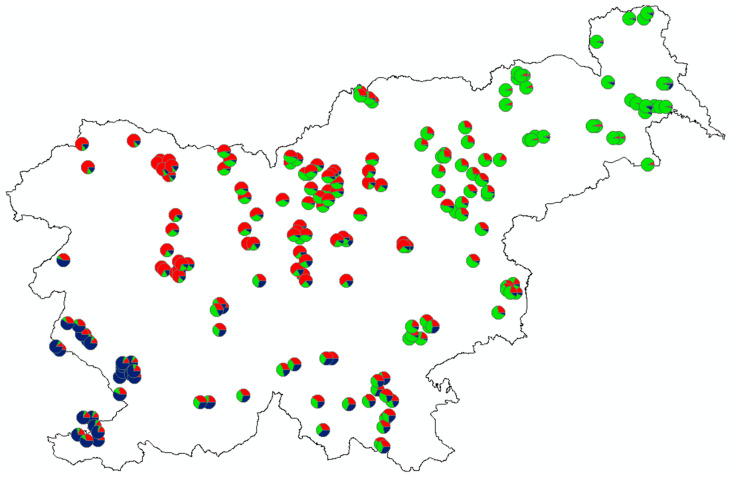
Genetic structure of roe deer in Slovenia based on spatial clustering of individuals according to the best model, dividing 10 populations into 3 clusters (K = 3; see also Figure 3).

The best model of population assignment obtained from the Structure (using ΔK according to [61]) separated individuals into three groups (K = 3), coinciding fairly well with their geographical origin (Figure 3 and Figure 4). Increasing in number of genetic clusters to K = 4 or K = 5 did not add any meaningful geographical structuring (Figure 3).

**Figure 5:**
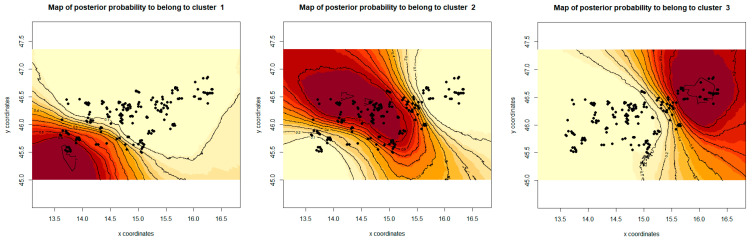
Maps showing the geographic distribution of samples (black points) with the relative posterior probability of belonging to each of the three inferred groups. The darker color reflects a higher posterior probability. Correlated allele frequencies model allowing for the presence of null alleles was calculated in Geneland v.4.9.2.

The correlated allele frequency model in Geneland also divided ten roe deer populations into three groups (Figure 5), clearly separating all southern populations from the northern/north-eastern ones, and showing similar results for the division as the Structure.

On the other hand, AMOVA did not strongly support the three-group structuring revealed by the Structure, DAPC, and Geneland, as the among-group variance was low and not significant. However, it nevertheless supported a significant association of individual’s genotype with its geographical position, as differences among populations within groups were significant (Table 3).

### 3.3. Isolation by Distance

**Figure 6:**
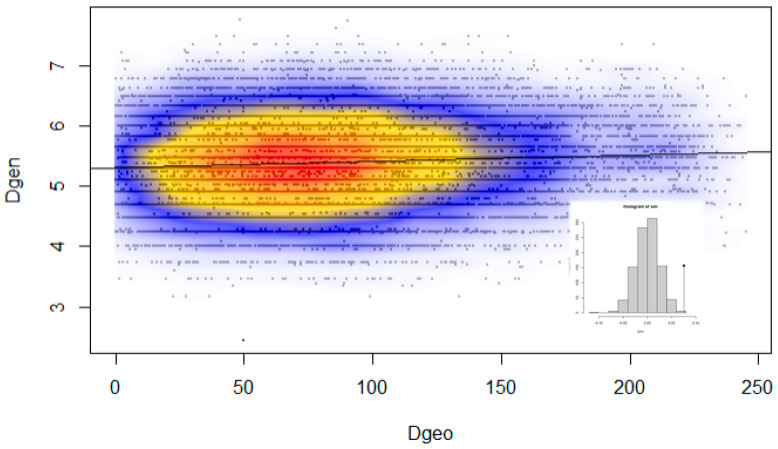
Pairwise Edwards genetic distances between individuals (Dgen), plotted against the Euclidean geographical distances (Dgeo; km) for the same individuals. Local density of points plotted using a two-dimensional Kernel density.

Microsatellite genetic distances in individuals were positively correlated with the geographical distances between them (*t* = 11.38, *p* < 0.001, *R*^2^ = 0.0056). Although this finding confirms to some extent that the geographical distance among individuals has some effect on the spatial genetic structure of roe deer in Slovenia, its influence is rather weak, explaining <1% of genetic variability among individuals (Figure 6).

### 3.4. Correlation between Individual Multilocus Heterozygosity and Fitness of Individuals

**Figure 7:**
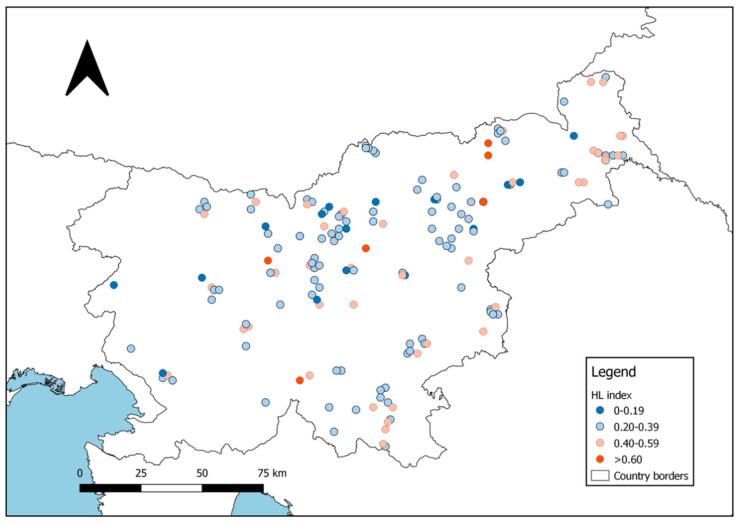
Individual multilocus heterozygosity (HL) of roe deer female yearlings included in the study.

The mean multilocus heterozygosity (HL index) of analyzed roe deer yearlings ranged from 0.00 to 0.69 (mean ± SD: 0.33 ± 0.13; for spatial distribution of individual HL values, see Figure 7). In the overall sample set collected throughout Slovenia, the relationship between the heterozygosity and standardized body mass of yearlings was significantly positive (*r* = 0.21; *p* < 0.001): 86% of individuals belonging to the highest HL class (≥0.60) were also in the highest body mass class (≥14.0 kg), and HL distribution of individuals in very poor body condition (<10.0 kg) was skewed towards the lower HL classes, with 9% in class 0.00–0.19, 82% in 0.20–0.39, 9% in 0.40–0.59, and none in ≥0.60 (compare Figure 7 with Figure S1 in Supplementary Material).

Apart from heterozygosity, some other intrinsic factors may also affect the fitness of individuals. Therefore, we tested the combined effect of HL and spatial genetic structure (i.e., membership in three groups/clusters defined by q-values for K = 3; see Figure 3) on the body mass of yearling females. After testing of all individual q-values for collinearity with HL, we excluded the proportion of membership for the second group (q2-value) of an individual’s assignment, and included q1- and q3-values into the linear regression models (Table 4). In these models, genetic traits explained up to 18.8% of body mass variability over the entire gradient of environmental factors faced by the species in Slovenia, and indicated that spatial genetic structure (particularly belonging to the genetic cluster q1, which is predominant in the North-eastern and Southern Slovenia; Figure 4) has a much stronger effect on roe deer body mass than heterozygosity (which only explains 3.0% of body mass variability).

The effect of heterozygosity on the reproductive ability of primiparous does was less pronounced than in the case of body mass. Nevertheless, all individuals belonging to the highest HL class (≥0.60) were fertile, i.e., they had ovulated (71% of them had two CL and 29% one CL), and HL distribution of non-fertile females was again skewed towards the lower HL classes, with 10% in the class 0.00–0.19, 70% in 0.20–0.39, 20% in 0.40–0.59, and none in ≥0.60 (compare Figure 7 with Figure S2 in Supplementary Material). In generalized linear models, which revealed that individual genetic characteristics significantly influenced both parameters of reproductive potential (Table 5), the heterozygosity effect is included in the best model for fertility (although its effect was not significant), but it is still the genetic component of each individual (belonging to the q1 cluster) that prevails. In the case of fertility, genetic features together with interannual variability (i.e., year of sampling) explain 19.2% of the variance. The effect of genetic factors (q1-value only) was less pronounced in the case of the potential reproductive output (the number of CL in fertile individuals), explaining only 4% of the variance.

## 4. Discussion

### 4.1. Genetic Diversity of Roe Deer in Slovenia

By utilizing microsatellite variations in European roe deer collected throughout Slovenia we determined the genetic variability and population structure of this species in the contact zone between the Alps and the Dinaric Mountains, i.e., in the wider area of the continent where data on the genetics of this the most common and widespread European wild ungulate have been completely lacking so far.

Across our sample set covering the whole distribution range of roe deer in the country, we found moderate microsatellite diversity (Ho = 0.57–0.65) in relation to the observed heterozygosity reported for other populations in Europe, where genetic diversity ranged from low (Ho = 0.17–0.58; [81]), to moderate (Ho = 0.63–0.66; [82]), to relatively high (Ho = 0.74–0.79; [83]). Genetic variability, expressed by allelic richness and heterozygosity indices, was higher in the central part of the country, and the lowest in Julian Alps and the Dinaric Mountains (Table 1). These areas are an unsuitable environment for roe deer, with dense old Dinaric forests (primarily Abieti-Fagetum) covering several tens of thousands of unbroken hectares along the Dinaric Mountains and a harsh alpine environment in the north of the country (Julian Alps), and the species has a low genetic diversity in this area, probably because its population densities are much lower in these regions ([14]; see Figure 1). This may, together with several geographic and anthropogenic barriers (seen particularly in the Alps in the form of alpine valleys and high mountains, rivers, and a highway), affect gene flow. Isolation and a significant effect from genetic drift likely contribute to lower allelic richness in these areas.

Apart from variability in recent population abundance, spatial differences in genetic diversity can also be explained by population history and/or different connectivity among subpopulations [84]. As far as historical data are concerned, roe deer populations which experienced genetic bottlenecks in the southern and northern (north-eastern) regions nevertheless retained a considerable amount of nuclear genetic diversity. This can be attributed to the ability of roe deer to expand very rapidly, thereby minimizing loss through genetic drift [85], but it is also due to the fact that in this species even a small number of founders and limited natural immigration would maintain high genetic diversity despite population differentiation [86]. Additionally, roe deer can recover rapidly in demographic terms, as the species is known to be ecologically adaptable and able to use a newly available habitat quickly [87]. This is because the species has a higher reproductive capacity than many other large mammals, as shown by an early age of first reproduction, regular production of twins [88], and relatively frequent litter sizes of three, and exceptionally even four and five, offspring [89].

Large contiguous populations with suitable habitat connectivity in central Slovenia tend to have higher genetic diversity compared to those from north-eastern agricultural areas (Table 1), where subpopulations have a smaller effective size, and individuals have lower need for long-distance migrations and roaming through agricultural land. There, the presence of large groups of roe deer (formerly known as the “field ecotype”; [90]; but see [39]), which often function as relatively closed units and are as such more isolated [91], may also affect gene flow: It is expected to be more intense within than among these large groups. However, previous comparative studies from other European countries did not find any genetic differentiation between roe deer inhabiting open fields and forested areas (e.g., seven countries of Central Europe [39]; North-eastern Poland [92]; Lithuania [93]). This suggests that individuals inhabiting the same areas, whether in forest or open habitats, are closely related, i.e., they do not represent two distinct ecotypes with a particular genetic integrity [39], and the ecological differences between them (for example, in social organization; reviewed in [25]) have appeared due to recent evolutionary separation from the common ancestor [93]. Nevertheless, socio-ecological differences between forest and field roe deer reflect the high behavioural plasticity of the species [25,39], including distinct reproductive behaviour (i.e., frequent matting excursions of females in forest-dwelling roe deer [94] that potentially mediate higher gene flow), which is in line with our presumption as to why the genetic diversity of roe deer is lower in the north-eastern, predominantly agricultural area of Slovenia, than in the central part of the country.

Our study revealed a heterozygote deficiency in almost all populations (except C5 and N1) with F_IS_ values ranging from −0.061 to 0.078. Although the effect of sample variance may have an impact on the results for populations C2 and N4, where sample sizes were smaller, and can therefore have a strong influence on parameter estimation, a possible explanation for this deficit could also be the presence of the Wahlund effect, which occurs when a spatial or temporal substructure is present in the sampled population [95]. In a temporal context, we used a very homogenous sample set of three successive years during which any changes of the genetic structure within populations would be almost impossible to detect. On the other hand, two out of three populations with significantly positive F_IS_ value (Table 1) were from areas where it had been either difficult to separate/classify animals a priori appropriately (C2) or where a population extends over an otherwise homogenous, but very wide area (C3); in both cases, we can expect a spatial impact and sub-structuring of the data within these populations (see Figure 1). In the N4 population living in the open agriculture area, however, the reason for a higher inbreeding coefficient might also be in the different social structure of roe deer. Our presumption is that the lower genetic diversity in this part of Slovenia could be connected to the presence of large, but from a gene flow point of view more closed, groups, representing a unique sub-structuring of roe deer in the open landscape.

### 4.2. Genetic Differentiation among Slovene Roe Deer Populations

The global F_ST_ value for the Slovenian roe deer is low (0.017) compared to populations examined in different European countries: 0.783 in Italy [81], 0.704 in Austria [83], and 0.038 in Switzerland [22]. However, low F_ST_ values in our populations are understandable considering the relatively small country size (although the whole of Slovenia was included), but also in view of the population history.

Levels of genetic differentiation vary among populations as shown by the F_ST_ comparisons (Table 2). Differences in 26 out of the 45 pairwise comparisons of F_ST_ were significant. The highest F_ST_ values were observed between the Julian Alps population (C1) vs. one of the northern (N3–Podravje and Slovenske gorice) and the southern population (S1–Coastal Slovenia), revealing an isolated population structure of roe deer in this part of the Alps. As populations S1 and C1 are geographically relatively close to each other (Figure 1), it seems obvious that for roe deer natural barriers—in this case between mountains and the coastal region—are sometimes an important factor limiting gene flow [41]. Similarly, it has been suggested that areas at high altitudes inhibit gene flow between red deer populations [96], which is likely to be an important factor for the isolation of the roe deer in the Slovene Julian Alps as well. Significant differences in F_ST_ were also observed between the population C5 (Posavsko Hills) and the closest northern population (N2; sub-Pannonian Slovenia). This shows differentiation in the absence of pronounced natural geographical features and could be related to the presence of anthropogenic barriers, such as urban areas [97] and particularly the highway dividing both populations (see Figure 1). The influence of transportation infrastructure on genetic divergence and discontinuities in roe deer leading to population differentiation has already been confirmed in some other European countries [22,23,98].

We found a signal for the existence of isolation by distance (Figure 6), especially when separating samples from the south (coastal Slovenia) from other populations. This is consistent with strong philopatry of roe deer females [25,84,99], inhibition of gene flow due to the altitude effect [96], and the presence of anthropogenic barriers (primarily the highway, built in the 1970s), which have prevented faster natural expansion of roe deer from Central Slovenia to the south and vice-versa. There is also a differentiation within the northern/north-eastern population group, which show genetic distances larger than in the central populations group. It is possible that this pattern reflects a combination of both natural (historical) and anthropogenic processes with differentiation according to distance. Taken together, the data suggest that central populations have been expanding south, north, and north-east, forming founder populations and thereafter meeting and reproducing with previously existing small residual groups (colonies) of roe deer there, which maintains relatively high genetic diversity in these areas despite obvious population differentiation (see also [86]). It is also possible that habitat fragmentation in more urbanized and for roe deer less suitable coastal habitats contributed to the higher F_ST_ values found within this area. Although the overall picture poses several puzzles regarding genetic diversity distribution and differentiation, the results are consistent across all the tests and support regional differentiation of Slovene roe deer populations as described above.

Wide-ranging habitat generalists such as different deer species are expected to exhibit low levels of population structure and a high potential for gene flow [100]. In our case, the evolution of population structure has been facilitated by the apparent historical extirpation of roe deer from much of their natural range (areas in the north-east and south), together with the existence of a relatively undisturbed remnant population (in the central part). Due to these historical features and the recent fast expansion of the species, we can see three main and pronouncedly differentiated genotype groups of roe deer in Slovenia (Figure 4), which may overlap. The existing admixture among them is reflected by a weak pattern of isolation by distance and slightly different results for the divisions of the populations (especially central ones) obtained by Geneland and DCPA vs. Structure. In contrast, with AMOVA we did not detect any differentiation between the groups recognized by previous spatial analyses. The absence of genetic differences among groups analyzed by AMOVA suggests that gene flow among them is taking place, and a significant molecular variance among populations within these three main clusters also suggests regional structuring of (sub)populations.

A large-scale, country-wide pattern in the genetic structure of roe deer in Slovenia is clearly defined by separation of the populations in the southern sub-Mediterranean and Karst regions as well as in the sub-Pannonian Region in the north-east from the populations in Central Slovenia, which is primarily due to historical reasons [11,12]. The small-scale genetic structure within regions is, however, a consequence of natural processes and indicates a strong tendency of the species towards philopatry with small home ranges and relatively low dispersal potential. Indeed, roe deer have rather small home ranges (often <<100 ha), especially in fragmented habitats [19,98,101], and dispersal of juveniles (yearlings) very rarely exceeds a few km [1]. After dispersal, roe deer show a high site fidelity, interrupted only occasionally by relatively short excursions in terms of distance of less than a few kilometers [1,102], for example as reproductive excursions [94]. In accordance with this, our data suggest a very limited dispersal of roe deer, which has been recently confirmed by genetic analysis of paternity over a 2400-ha-wide area typical for Central Slovenia [41]. These results are also consistent with previous genetic studies of the species in Europe, in which roe deer were confirmed to be highly sedentary and showed little or no evidence for sex-biased dispersal [19,103,104]. For example, a study based on the fine-scale genetic structure of roe deer in France revealed that the spatial distribution of individuals was not random: Adults of both sexes were usually located close to their relatives [104]. A high tendency for site fidelity of the species is an obvious further restriction on movement of roe deer in a fragmented landscape: In highly fragmented habitats there is a more pronounced correlation between genetic distance and urbanization than with geographical distance [97]. Therefore, the expected high degree of isolation by distance generated by high site fidelity, philopatry, and the short-range dispersal potential of roe deer may be further disturbed (i.e., facilitated) by habitat fragmentation, as can be observed in Europe due to agricultural practices or fragmentation of forests [105], and construction of long fences along infrastructure [19,22,23] or, more recently, along country borders [106].

### 4.3. Correlation between Genetic Traits and Fitness of Roe Deer

Roe deer is a typical income breeder with few available body reserves [29]. Therefore, its body mass shows relatively low seasonality; for example, adult males experience much lower rut-related loss of body mass (7.5%) than their counterparts in capital breeders, such as red deer or chamois [30]. In consequence, roe deer body mass is a relevant proxy for an individual’s condition and phenotypic quality [107]. However, contrary to the different environmental factors known to influence body mass across the whole native distribution range of roe deer (e.g., [108,109,110]), the influence of intrinsic (genetic) factors has been very rarely studied and previous results are controversial. Therefore, in addition to defining the genetic outlook and structure of roe deer we also aimed to analyze whether genetic advantage (expressed as higher heterozygosity) together with other genetic data (reflected in the structuring of the population) has any influence on the body mass of studied individuals.

We found that genetic factors have a significant influence on the body mass of roe deer yearling females, explaining 18.8% of its variance. By previous determination of the population’s genetic structure, we were able to divide the genetic effect into two components, the first one connected with a genetic vigour (heterozygosity) and the second one with a spatial genetic context (structuring of populations). A level of individual heterozygosity alone significantly positively affected body mass, which is in accordance with the theory that heterozygosity is commonly positively correlated with fitness in wild populations [111]. This is also in line with the finding that heterozygous juveniles have higher fitness early in life because they develop faster and survive better [112,113], and with previous studies of adult white-tailed deer (*Odocoileus virginianus*) in Finland, where Brommer et al. [114] confirmed that individual heterozygosity positively correlated with age- and sex-corrected body mass (but did not have an effect on jaw size or antler quality). Similarly, Brambilla et al. [115] found that standardized multi-locus heterozygosity was related to body mass and horn growth in Alpine ibex (*Capra ibex*) at Gran Paradiso National Park, Italy. However, all these results are in contrast to findings that the heterozygous advantage disappears after weaning because thereafter higher heterozygosity is no longer associated with better body condition or the dispersal ability of different vertebrates [116,117]. In roe deer from three populations in France (both sexes included), Vanpé et al. [116] found that heterozygosity was not correlated with body mass at the age of eight months (at the end of the maternal care period) and they did not find any positive effect of heterozygosity on dispersal propensity or distance. They put the absence of a correlation between heterozygosity and body mass at this age down to the fact that viability selection in the early stages of life, which in roe deer occurs in the first months of life, leads to high natal mortality [118,119]. However, in the cultural landscape post-natal mortality of roe deer fawns is strongly dependent on non-viability-related factors such as red fox (*Vulpes vulpes*) predation, mowing, and other agricultural machinery operations [120,121,122,123], which also holds true in Slovenia [124]. Therefore, we would expect that viability selection in roe deer fawns is not that pronounced and that the ratio of viable vs. non-viable individuals that survive from fawns to yearlings would not be completely skewed towards viable ones. Moreover, in our study we operated with one sex only and had the advantage of preselecting samples for genetic analyses from a large sample set of females that had been previously analyzed for their reproductive ability [33], hence giving us better insight into their viability. Consequently, we included in the study specimens across a large range of body masses (8.0–20.5 kg measured at the harvest, and 7.4–21.2 kg after standardization, respectively), covering individuals in poor, average, and good body conditions. We believe that due to this and contrary to Vanpé et al. [116], there is a slight, but significant effect of heterozygosity on the body mass of roe deer female yearlings.

Despite its statistically significant effect which is particularly clear at both extremes (the majority of individuals with the highest HL index were in good body condition (>14.0 kg); 91% of individuals in poor body condition (<10 kg) had HL < 0.40), heterozygosity alone explained <5% of the total body mass variance. A more important genetic driver was connected with the population structure, particularly with the probability of being a member of the first genetic cluster (q1). Individuals with a higher q1-value (Figure 4, circles with predominant green color) prevailed in North-eastern (sub-Pannonian) Slovenia where they face better living conditions (a larger proportion of open agriculture areas, moderate continental climate). There, roe deer have on average higher body mass compared to their forest counterparts from other areas [35]; in female yearlings, differences are as much as 0.5–1.5 and 1.5–2.2 kg in comparison with the Alpine/pre-Alpine and sub-Mediterranean regions, respectively [125]. This is in line with general findings that the body mass of roe deer (particularly yearlings) is strongly dependent on environmental conditions causing variability in available resources [108,109,110]. Therefore, considering geographic differences in habitat suitability for roe deer in Slovenia (low to high gradient in south-western to north-eastern direction) which coincide with the changes in roe deer population genetic structure expressed by the membership in one of the three recognised clusters (increase of q1-value and decrease of q-3 value in the south-western to north-eastern gradient), we were not able to claim that the genetic membership probability per se hafd an effect on body mass variability. As the genetic diversity of roe deer in North-eastern Slovenia is lower than in large contiguous populations with suitable habitat connectivity in the central part of the country, it seems that environmental factors determining the availability of (food) resources—and geographically coinciding with the population structuring—prevail over the effect of genetic factors.

The effect of genetic factors on another important component of fitness, reproductive ability, was less pronounced. Although heterozygosity was included in all the best models for both fertility (ovulated vs. non-ovulated individuals) and the potential reproductive outcome (number of corpora lutea per ovulating female), and its meaning is evident at both extremes (all individuals with the highest HL index had ovulated, 71% of them carried two CL; 80% of non-fertile yearlings had HL < 0.40), we could not confirm its significant effect on either of the two tested parameters. However, the population structure (the probability of being a member of the first genetic cluster; q1) had a significant effect both on fertility and on potential reproductive outcome (Table 5). But in this case the result could be strongly influenced by the overlap of the population genetic structure with the gradient of environmental factors (see above), either directly or indirectly, through their effects on body mass, which is the main factor influencing the reproductive ability of roe deer females, particularly yearlings [32,33]. That the effect of environmental factors on fertility prevails over genetic ones indicates the fact that the year of sampling (2013, 2014, and 2015) was recognized as a significant covariate with much stronger effect that any of genetic factors (Table 5). In those study years, high interannual variability in the reproductive potential of roe deer females had already been revealed by Flajšman and Pokorny [126] who found that 83% of primiparous does ovulated in 2013, and had in their ovaries on average 1.2 CL; these figures were much lower compared to either 2014 (97%; 1.6) or 2015 (98%; 1.5). Authors explained that this interannual variability was due to differences in weather conditions between years, with a much hotter and dryer summer in 2013 (mean July data: Temperature of 20.4 °C; rainfall of 47 mm) than in 2014 (18.2 °C; 167 mm). This again indicates that although genetic factors have some effect on the fitness of roe deer, this is either masked or overruled by the influence of environmental factors. Therefore, more research on the holistic effect of external and intrinsic factors on selected parameters of fitness and other life-history traits in roe deer are needed to better understand the real influence of genetics on evolution and the expected population dynamics of this key terrestrial species.

## 5. Conclusions

In Slovenia, the roe deer has developed a pronounced population structure in accordance with geographic features. Such a population genetic structure confirms the high side fidelity of the species, but there are nevertheless admixtures of genes among different areas (populations). Although the genetics have an influence on the fitness/vitality of individuals, this is predominantly determined by environmental factors, i.e., availability of resources. From the perspective of applied evolutionary inference, this study illustrates how important it is to understand the holistic effects of genetic and environmental factors on life-history traits when adaptive, science-based population management is required, and when decisions are based on monitoring of fitness parameters.

## Figures and Tables

**Table 1:** Genetic diversity in Slovenian roe deer populations based on 11 microsatellite loci.

Geographical Area	Abbr.	n	He ± SD	Ho ± SD	F_IS_	HWE (*p*-Values)	A ± SD	AR ± SD
Coastal Slovenia (Kras and Istra)	S1	33	0.669 ± 0.139	0.655 ± 0.145	0.021	0.157	6.273 ± 2.494	4.817 ± 1.804
Julian Alps	C1	10	0.610 ± 0.154	0.646 ± 0.216	−0.061	0.286	4.455 ± 1.968	4.455 ± 1.968
Polhograjsko and Škofjeloško Hills	C2	15	0.656 ± 0.155	0.610 ± 0.157	**0.073**	0.500	5.455 ± 2.115	4.973 ± 1.836
Dinaric Mountains	C3	27	0.623 ± 0.171	0.577 ± 0.184	**0.075**	0.569	5.545 ± 2.207	4.506 ± 1.747
Kamniško-Savinjske Alps	C4	34	0.652 ± 0.160	0.642 ± 0.204	0.015	0.326	6.545 ± 2.659	5.013 ± 1.786
Posavsko Hills	C5	16	0.630 ± 0.139	0.640 ± 0.131	−0.017	0.629	5.364 ± 2.292	4.738 ± 1.901
Pohorje	N1	21	0.622 ± 0.185	0.641 ± 0.207	−0.031	0.802	5.636 ± 2.501	4.683 ± 1.967
Sub-Pannonian Region	N2	25	0.629 ± 0.174	0.618 ± 0.210	0.017	0.555	6.000 ± 2.490	4.865 ± 1.951
Podravje and Slovenske gorice	N3	16	0.632 ± 0.250	0.611 ± 0.239	0.035	0.013	5.364 ± 2.767	4.782 ± 2.423
Prekmurje	N4	17	0.618 ± 0.201	0.571 ± 0.222	**0.078**	0.566	5.273 ± 2.37	4.658 ± 1.949

Notes: Standard deviations (SD) are for average values per locus; significant values are indicated in bold; He: Expected heterozygosity, Ho: Observed heterozygosity, F_IS_: Inbreeding coefficient, HWE: Hardy–Weinberg equilibrium, A: Number of alleles, AR: Allelic richness (calculated by the rarefaction method for the lowest sample size *n* = 10).

**Table 2:** Pairwise values of F_ST_ among ten roe deer populations in Slovenia.

Population	S1	C1	C2	C3	C4	C5	N1	N2	N3
C1	**0.007**								
C2	0.033	**0.012**							
C3	0.011	**0.006**	0.022						
C4	0.020	**−0.002**	**0.012**	0.006					
C5	0.024	**0.000**	0.024	**0.012**	**0.012**				
N1	0.030	0.019	0.028	**0.001**	**−0.002**	0.021			
N2	0.020	**0.008**	**0.013**	**0.004**	**−0.001**	**0.012**	**0.002**		
N3	0.040	0.040	0.031	0.019	0.017	0.035	**0.008**	**0.006**	
N4	0.028	0.026	0.033	0.022	0.032	0.038	0.024	0.018	**0.009**

Values in bold are not significant, indicating that we did not find differences in F_ST_ between these pairs of comparisons.

**Table 3:** Hierarchical analysis of molecular variance (AMOVA) based on microsatellite data.

Source of Variation	Variance
Within individuals	**94.92** (<0.0004)
Among individuals within populations	**3.39** (<0.008)
Among populations within groups	**1.20** (<0.0001)
Among groups	0.48 (<0.103)

Notes: Values in bold are significant. The populations correspond to ten populations predefined by the geographical position of harvested individuals (see Table 1), and groups correspond to the three clusters according to the results of the Geneland and Structure analysis (see Figure 2, Figure 3 and Figure 4); *p*-values are given in parenthesis.

**Table 4:** Linear regression models, showing the influence of genetic traits on body mass of yearling roe deer females in Slovenia. The explanatory variables are heterozygosity and membership coefficients (q1- and q3-values) of individuals.

Model 1 (The Best Model): *p* < 0.001, *R*^2^ = 0.188				
Variable	Estimate	Standard Error	*t*-Value	*p*-Value
Intercept	14.6247	0.5768	25.355	<0.001
HL	2.6683	1.2856	2.076	0.039
q1	3.8706	0.7453	5.193	<0.001
q3	3.0237	1.1870	−2.547	0.012
**Model 2: *p* < 0.001, *R*^2^ = 0.116**				
Intercept	14.1964	0.5608	25.314	<0.001
HL	2.9129	1.3030	2.235	0.027
q1	3.9410	0.7570	−5.206	<0.001
**Model 3: *p* = 0.002, *R*^2^ = 0.061**				
Intercept	13.0893	0.5326	24.576	<0.001
HL	3.2131	1.3779	2.332	0.021
q3	3.2522	1.2756	−2.549	0.012
**Model 4: *p* = 0.014, *R*^2^ = 0.030**				
Intercept	12.5979	0.5046	24.965	<0.001
HL	3.4872	31.3961	2.498	0.013

**Table 5:** Generalized linear models of fertility and potential reproductive output of yearling roe deer females in Slovenia (period 2013–2015). The independent variables were HL (covariate), q-values, and year of sampling (a fixed factor only in fertility analysis). Model selection was performed using the Akaike information criteria (AIC). The best models and other models with ΔAIC <2 are shown.

Fertility				
Best Model: AIC = 74.65; *R*^2^ = 0.192				
Variable	Estimate	Standard Error	*t*-Value	*p*-Value
Intercept	−2769.2212	950.8732	−2.912	0.003
HL	1.4502	2.5843	0.561	0.575
q1	5.0109	2.1728	−2.306	0.021
q3	16.7065	12.3516	1.353	0.176
year	1.3764	0.4723	2.914	0.004
**Potential Reproductive Output**				
**Best Model: AIC = 212.3; *R*^2^ = 0.037**				
Intercept	0.6676	0.5435	1.228	0.219
HL	0.9163	1.2725	0.720	0.471
q1	−1.6956	0.7565	−2.241	0.025
**Model 2: AIC = 214.2; *R*^2^ = 0.038**				
Intercept	0.7179	0.5721	1.255	0.209
HL	0.8885	1.2765	0.696	0.486
q1	−1.6916	0.7579	−2.232	0.026
q3	−0.3268	1.1401	−0.287	0.774

The bold values are significant p values below 0.05. It is common use to put this values in bold.

## Supplementary Materials

The following are available online at https://www.mdpi.com/2076-2615/10/12/2276/s1, Figure S1: Standardized body mass of roe deer female yearlings included in the study, Figure S2: Number of corpora lutea (CL) in ovaries of roe deer yearlings included in the study, Table S1: Basic data on roe deer females, included in the study of genetic structure and heterozygosity–fitness relations in Slovenia. Table S2: List of microsatellite loci used in the population genetic analysis of *Capreolus capreolus*. Table S3: The null alleles frequency estimates by three different programmes.
