# Supplementary material for: First Insights into the Population Genetic Structure and Heterozygosity–Fitness Relationship in Roe Deer Inhabiting the Area between the Alps and Dinaric Mountains
Source: Animals (Basel). 2020 Dec 2;10(12):2276. doi: 10.3390/ani10122276 (PMC7761463; doi:10.3390/ani10122276)
Supplement: Supplementary file 1 [file animals-10-02276-s001.pdf]

Article

First Insights into the Population Genetic Structure and Heterozygosity–Fitness Relationship in Roe Deer Inhabiting the Area between the Alps and Dinaric Mountains

Elena Buzan [1,2], Urška Gerič [1], Sandra Potušek [1], Katarina Flajšman [3] and Boštjan Pokorny [2,3,*]

[1] Natural Sciences and Information Technologies, Faculty of Mathematics, University of Primorska, Glagoljaška 8, 6000 Koper, Slovenia; elena.buzan@upr.si (E.B.), urska.geric@famnit.upr.si (U.G.), sandra.potusek@famnit.upr.si (S.P.)
[2] Environmental Protection College, Trg mladosti 7, 3320 Velenje, Slovenia; bostjan.pokorny@vsvo.si
[3] Slovenian Forestry Institute, Večna pot 2, 1000 Ljubljana, Slovenia; katarina.flajsman@gozdis.si
* Correspondence: bostjan.pokorny@vsvo.si

Received: 23 October 2020; Accepted: 28 November 2020; Published: date

Supplementary material

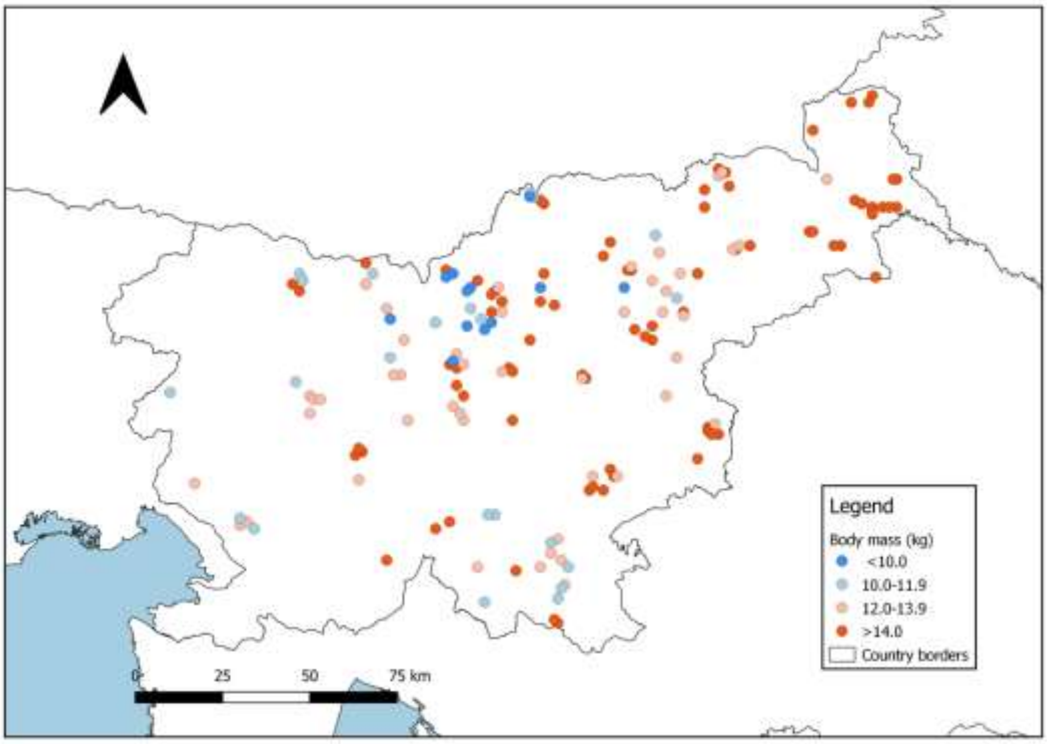

Figure S1. Standardized body mass of roe deer female yearlings included in the study.

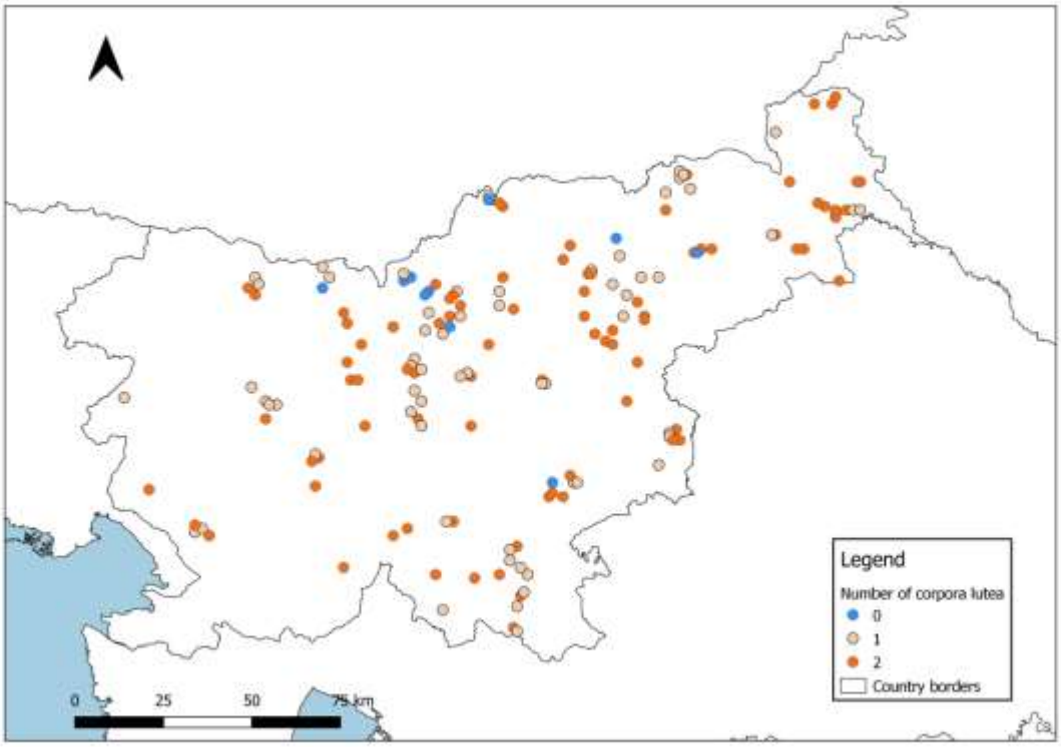

Figure S2. Number of corpora lutea (CL) in ovaries of roe deer yearlings included in the study.

animals

MDPI

Table S1. Basic data on roe deer females, included in the study of genetic structure and heterozygosity–fitness relations in Slovenia. All individuals were sampled in the period 2013–2015 in 58 hunting grounds, continuously distributed throughout Slovenia (see Fig. 1), and were included in determination of population genetic structure. For assessment of the effects of genetic features on fitness, only yearlings were employed.

	Area	Population	Latitude	Longitude	Date of harvest	Age class	Body mass at harvest (kg)	Standardized body mass (kg)	Number of corpora lutea (CL)	HL value
LME134	Coastal Slovenia (Kras and Istra)	S1	46.308	14.430	18 Oct 2014	Yearling	14.5	13.41	2	0.390
LME139	Coastal Slovenia (Kras and Istra)	S1	46.073	14.161	25 Sep 2014	Yearling	13	12.57	1	0.212
LME140	Coastal Slovenia (Kras and Istra)	S1	46.396	14.104	16 Sep 2014	Yearling	12.5	11.93	1	0.000
LME1428	Coastal Slovenia (Kras and Istra)	S1	46.378	14.118	29 Sep 2014	Adult	16	16.41	2	0.515
LME143	Coastal Slovenia (Kras and Istra)	S1	46.354	14.729	29 Sep 2014	Yearling	12.5	12.13	1	0.463
LME1432	Coastal Slovenia (Kras and Istra)	S1	46.228	14.496	6 Oct 2014	Adult	19	18.73	0	0.231
LME1433	Coastal Slovenia (Kras and Istra)	S1	46.192	14.691	29 Oct 2014	Adult	12	15.08	2	0.534
LME1438	Coastal Slovenia (Kras and Istra)	S1	46.229	14.963	29 Oct 2014	Adult	13.5	13.58	2	0.338
LME1439	Coastal Slovenia (Kras and Istra)	S1	46.381	14.768	26 Sep 2014	Adult	17	16.58	2	0.251
LME1440	Coastal Slovenia (Kras and Istra)	S1	46.300	14.820	3 Nov 2014	Adult	13.5	13.65	1	0.319
LME1441	Coastal Slovenia (Kras and Istra)	S1	46.255	14.794	23 Oct 2014	Adult	13.5	13.46	2	0.493
LME1442	Coastal Slovenia (Kras and Istra)	S1	46.174	14.678	25 Nov 2014	Adult	16	16.49	1	0.312
LME1443	Coastal Slovenia (Kras and Istra)	S1	46.156	14.691	25 Sep 2014	Adult	14	13.57	2	0.430
LME1444	Coastal Slovenia (Kras and Istra)	S1	46.165	14.717	5 Oct 2014	Adult	12	11.72	2	0.136
LME1445	Coastal Slovenia (Kras and Istra)	S1	46.363	14.742	24 Sep 2014	Adult	14	13.55	1	0.299
LME1449	Coastal Slovenia (Kras and Istra)	S1	46.301	14.859	21 Dec 2014	Adult	17	17.88	2	0.251
LME1450	Coastal Slovenia (Kras and Istra)	S1	46.309	14.742	17 Dec 2014	Adult	18	18.82	2	0.363
LME1454	Coastal Slovenia (Kras and Istra)	S1	46.400	15.015	27 Dec 2014	Adult	19.5	20.47	2	0.159
LME1455	Coastal Slovenia (Kras and Istra)	S1	46.273	14.820	23 Dec 2014	Adult	20	20.91	2	0.351
LME1459	Coastal Slovenia (Kras and Istra)	S1	45.509	15.053	21 Dec 2014	Adult	15.5	16.38	2	0.362
LME1460	Coastal Slovenia (Kras and Istra)	S1	45.644	15.002	30 Nov 2014	Adult	14	14.56	2	0.670
LME1466	Coastal Slovenia (Kras and Istra)	S1	45.680	15.040	23 Dec 2015	Adult	18	18.91	2	0.240
LME1467	Coastal Slovenia (Kras and Istra)	S1	45.662	15.079	18 Dec 2015	Adult	20	20.84	2	0.239
LME1468	Coastal Slovenia (Kras and Istra)	S1	45.779	14.809	5 Nov 2015	Adult	17	17.19	2	0.221
**Sample ID**	**Area**	**Population**	**Latitude**	**Longitude**	**Date of harvest**	**Age class**	Body mass at harvest (kg)	**Standardized body mass (kg)**	**Number of corpora lutea (CL)**	**HL value**
LME1469	Coastal Slovenia (Kras and Istra)	S1	45.707	15.040	24 Dec 2015	Adult	20	20.93	2	0.238
LME1470	Coastal Slovenia (Kras and Istra)	S1	45.948	14.331	4 Oct 2015	Adult	20	19.70	2	0.387
LME1471	Coastal Slovenia (Kras and Istra)	S1	46.021	14.511	26 Sep 2015	Adult	17	16.58	2	0.340
LME1472	Coastal Slovenia (Kras and Istra)	S1	45.641	14.245	6 Sep 2015	Adult	19	18.28	2	0.356
LME1473	Coastal Slovenia (Kras and Istra)	S1	45.642	14.283	6 Sep 2015	Adult	17	16.28	2	0.308
LME1477	Coastal Slovenia (Kras and Istra)	S1	45.660	14.437	-	-	11.5	-	2	0.330
LME252	Coastal Slovenia (Kras and Istra)	S1	45.537	13.696	6 Sep 2015	Yearling	13	12.28	2	0.315
LME253	Coastal Slovenia (Kras and Istra)	S1	45.520	13.786	8 Sep 2015	Yearling	12	11.31	2	0.237
LME254	Coastal Slovenia (Kras and Istra)	S1	45.835	13.740	26 Sep 2015	Yearling	11	10.58	2	0.138
LME1474	Julian Alps	C1	45.592	13.720	-	-	13.5	-	2	0.193
LME1475	Julian Alps	C1	45.565	13.772	-	-	15.5	-	2	0.309
LME1476	Julian Alps	C1	46.658	15.681	18 Sep 2015	Adult	17	16.46	2	0.311
LME1478	Julian Alps	C1	45.641	14.245	-	-	16	-	2	0.342
LME1479	Julian Alps	C1	45.930	14.318	-	-	15	-	2	0.564

LME273	Julian Alps	C1	45.867	14.332	2 Sep 2015	Yearling	12	11.22	1	0.267
LME274	Julian Alps	C1	45.779	14.834	3 Sep 2015	Yearling	15	14.23	2	0.409
LME275	Julian Alps	C1	45.644	14.771	5 Sep 2015	Yearling	15	14.26	2	0.365
LME276	Julian Alps	C1	45.592	13.720	4 Sep 2015	Yearling	11.5	10.47	1	0.334
LME334	Julian Alps	C1	45.547	13.785	13 Sep 2015	Yearling	8	7.38	0	0.159
LME122	Polhograjsko and Škofjeloško hills	C2	46.045	14.136	17 Sep 2014	Yearling	13.5	12.94	2	0.248
LME123	Polhograjsko and Škofjeloško hills	C2	46.046	14.149	13 Oct 2014	Yearling	12	11.84	2	0.619
LME124	Polhograjsko and Škofjeloško hills	C2	46.055	14.149	18 Sep 2014	Yearling	13	13.46	2	0.581
LME1446	Polhograjsko and Škofjeloško hills	C2	46.165	14.665	2 Sep 2014	Adult	17	16.29	2	0.222
LME1447	Polhograjsko and Škofjeloško hills	C2	46.282	14.781	3 Sep 2014	Adult	17	17.06	2	0.312
LME1461	Polhograjsko and Škofjeloško hills	C2	45.590	15.079	17 Sep 2014	Adult	14	13.80	2	0.480
LME1462	Polhograjsko and Škofjeloško hills	C2	45.563	15.066	3 Sep 2014	Adult	14	13.23	2	0.436
LME1463	Polhograjsko and Škofjeloško hills	C2	45.716	15.066	3 Sep 2014	Adult	13	12.23	3	0.389
LME1464	Polhograjsko and Škofjeloško hills	C2	45.742	14.616	1 Oct 2014	Adult	14	13.66	2	0.239
**Sample ID**	**Area**	**Population**	**Latitude**	**Longitude**	**Date of harvest**	**Age class**	Body mass at harvest (kg)	**Standardized body mass (kg)**	**Number of corpora lutea (CL)**	**HL value**
LME1465	Polhograjsko and Škofjeloško hills	C2	45.760	14.667	6 Sep 2014	Adult	15.5	14.78	2	0.248
LME189	Polhograjsko and Škofjeloško hills	C2	46.589	15.002	6 Sep 2014	Yearling	13.5	12.78	2	0.233
LME230	Polhograjsko and Škofjeloško hills	C2	46.564	16.241	17 Oct 2015	Yearling	11.5	11.40	1	0.176
LME231	Polhograjsko and Škofjeloško hills	C2	46.834	16.234	17 Oct 2015	Yearling	13	12.90	1	0.378
LME236	Polhograjsko and Škofjeloško hills	C2	46.852	16.248	20 Oct 2015	Yearling	13.5	13.44	1	0.424
LME237	Polhograjsko and Škofjeloško hills	C2	46.834	16.168	12 Oct 2015	Yearling	13.5	13.32	1	0.380
LME125	Dinaric Mountains	C3	46.408	15.327	20 Dec 2014	Yearling	19	19.87	2	0.536
LME126	Dinaric Mountains	C3	46.444	15.236	5 Nov 2014	Yearling	14	14.19	1	0.234
LME136	Dinaric Mountains	C3	46.425	14.351	25 Oct 2014	Yearling	14	14.02	2	0.555
LME137	Dinaric Mountains	C3	46.396	14.065	26 Sep 2014	Yearling	14.5	14.41	1	0.333
LME142	Dinaric Mountains	C3	46.290	15.534	15 Sep 2014	Yearling	14	13.41	2	0.312
LME1451	Dinaric Mountains	C3	46.658	15.694	15 Sep 2014	Adult	17	18.03	2	0.430
LME1452	Dinaric Mountains	C3	46.569	15.615	26 Dec 2014	Adult	16	16.96	2	0.652
LME1453	Dinaric Mountains	C3	46.614	15.615	31 Dec 2014	Adult	17	18.03	2	0.432
LME164	Dinaric Mountains	C3	46.117	14.096	5 Sep 2014	Yearling	14	13.26	2	0.471
LME165	Dinaric Mountains	C3	46.073	14.187	6 Sep 2014	Yearling	12	11.28	2	0.450
LME166	Dinaric Mountains	C3	46.369	14.079	1 Sep 2014	Yearling	12	11.20	1	0.426
LME168	Dinaric Mountains	C3	46.363	14.846	6 Sep 2014	Yearling	14	13.28	2	0.212
LME175	Dinaric Mountains	C3	46.354	14.833	20 Oct 2014	Yearling	14.5	14.44	2	0.666
LME176	Dinaric Mountains	C3	46.345	14.820	20 Sep 2014	Yearling	15	14.49	2	0.444
LME318	Dinaric Mountains	C3	46.650	15.668	13 Sep 2015	Yearling	15	14.38	2	0.460
LME319	Dinaric Mountains	C3	46.460	15.718	11 Sep 2015	Yearling	13	12.35	2	0.301
LME320	Dinaric Mountains	C3	46.469	15.744	2 Oct 2015	Yearling	16	15.67	2	0.336
LME321	Dinaric Mountains	C3	46.469	15.783	1 Sep 2015	Yearling	12	11.20	2	0.277
LME322	Dinaric Mountains	C3	46.460	15.731	8 Sep 2015	Yearling	14	13.31	2	0.206
LME323	Dinaric Mountains	C3	46.022	14.898	3 Sep 2015	Yearling	15	14.23	2	0.279
LME324	Dinaric Mountains	C3	46.148	14.898	5 Sep 2015	Yearling	12	11.26	1	0.598
LME325	Dinaric Mountains	C3	46.057	14.679	16 Sep 2015	Yearling	13	12.43	1	0.331
**Sample ID**	**Area**	**Population**	**Latitude**	**Longitude**	**Date of harvest**	**Age class**	Body mass at harvest (kg)	**Standardized body mass (kg)**	**Number of corpora lutea (CL)**	**HL value**
LME326	Dinaric Mountains	C3	46.084	14.717	5 Oct 2015	Yearling	11	10.72	1	0.360
LME81	Dinaric Mountains	C3	45.851	15.195	3 Oct 2013	Yearling	13	12.69	1	0.257
LME82	Dinaric Mountains	C3	45.877	15.272	6 Oct 2013	Yearling	13	12.73	1	0.369

LME93	Dinaric Mountains	C3	45.878	15.195	8 Oct 2013	Yearling	14	10.76	1	0.232
LME95	Dinaric Mountains	C3	45.895	15.259	18 Dec 2013	Yearling	13	11.84	1	0.221
LME104	Kamniško-Savinjske Alps	C4	46.371	14.352	7 Sep 2013	Yearling	14	12.29	0	0.309
LME105	Kamniško-Savinjske Alps	C4	46.398	14.377	1 Dec 2013	Yearling	11	11.58	1	0.452
LME106	Kamniško-Savinjske Alps	C4	46.390	14.651	16 Sep 2013	Yearling	9.5	8.93	0	0.544
LME107	Kamniško-Savinjske Alps	C4	46.399	14.677	18 Sep 2013	Yearling	10.5	9.96	0	0.323
LME108	Kamniško-Savinjske Alps	C4	46.273	14.612	26 Nov 2013	Yearling	14	14.50	0	0.326
LME109	Kamniško-Savinjske Alps	C4	46.417	15.340	16 Sep 2013	Yearling	14	13.43	1	0.269
LME113	Kamniško-Savinjske Alps	C4	46.606	14.963	2 Oct 2014	Yearling	16	15.67	2	0.673
LME144	Kamniško-Savinjske Alps	C4	46.137	14.458	6 Oct 2014	Yearling	11	10.73	2	0.363
LME145	Kamniško-Savinjske Alps	C4	46.273	14.612	11 Dec 2014	Yearling	15	15.73	1	0.352
LME146	Kamniško-Savinjske Alps	C4	46.264	14.729	14 Sep 2014	Adult	13.5	12.90	2	0.611
LME147	Kamniško-Savinjske Alps	C4	46.328	14.859	23 Dec 2014	Yearling	14	14.91	1	0.256
LME170	Kamniško-Savinjske Alps	C4	45.500	15.066	14 Sep 2014	Yearling	12	11.40	2	0.232
LME171	Kamniško-Savinjske Alps	C4	45.939	14.344	5 Sep 2014	Yearling	10.5	9.76	2	0.245
LME172	Kamniško-Savinjske Alps	C4	45.877	15.285	3 Sep 2014	Yearling	14	13.23	2	0.163
LME186	Kamniško-Savinjske Alps	C4	46.453	15.444	1 Dec 2014	Yearling	14.5	15.08	2	0.066
LME194	Kamniško-Savinjske Alps	C4	46.408	15.340	16 Sep 2014	Yearling	9	8.43	1	0.321
LME196	Kamniško-Savinjske Alps	C4	46.398	15.522	13 Oct 2014	Yearling	16.5	16.34	2	0.311
LME197	Kamniško-Savinjske Alps	C4	46.597	14.963	16 Sep 2014	Yearling	10	9.43	1	0.314
LME198	Kamniško-Savinjske Alps	C4	46.398	15.587	20 Sep 2014	Yearling	10.5	9.99	1	0.214
LME199	Kamniško-Savinjske Alps	C4	46.398	15.587	30 Sep 2014	Yearling	15.5	15.14	2	0.312
LME200	Kamniško-Savinjske Alps	C4	46.637	16.073	14 Oct 2014	Yearling	13	12.85	1	0.229
LME207	Kamniško-Savinjske Alps	C4	45.993	15.621	4 Sep 2014	Yearling	9	8.25	0	0.246
LME242	Kamniško-Savinjske Alps	C4	45.765	13.935	10 Nov 2015	Yearling	15	15.26	2	0.433
**Sample ID**	**Area**	**Population**	**Latitude**	**Longitude**	**Date of harvest**	**Age class**	Body mass at harvest (kg)	**Standardized body mass (kg)**	**Number of corpora lutea (CL)**	**HL value**
LME280	Kamniško-Savinjske Alps	C4	46.148	14.859	8 Sep 2015	Yearling	14	13.31	2	0.283
LME305	Kamniško-Savinjske Alps	C4	46.084	15.467	6 Sep 2015	Yearling	12	11.28	2	0.214
LME306	Kamniško-Savinjske Alps	C4	46.182	15.507	4 Sep 2015	Yearling	13	12.25	1	0.184
LME309	Kamniško-Savinjske Alps	C4	46.264	15.417	6 Sep 2015	Yearling	16	15.28	2	0.259
LME310	Kamniško-Savinjske Alps	C4	46.300	15.456	3 Sep 2015	Yearling	11.5	10.73	1	0.403
LME313	Kamniško-Savinjske Alps	C4	46.237	15.417	3 Oct 2015	Yearling	14	13.69	1	0.468
LME314	Kamniško-Savinjske Alps	C4	46.228	15.417	9 Sep 2015	Yearling	15	14.32	2	0.212
LME315	Kamniško-Savinjske Alps	C4	46.363	15.314	4 Sep 2015	Yearling	15	14.25	2	0.369
LME76	Kamniško-Savinjske Alps	C4	46.580	15.015	25 Oct 2013	Yearling	13.5	13.52	2	0.327
LME88	Kamniško-Savinjske Alps	C4	46.446	13.713	2 Nov 2013	Yearling	16	16.14	2	0.118
LME97	Kamniško-Savinjske Alps	C4	46.111	14.691	17 Sep 2013	Yearling	9	8.44	0	0.324
LME173	Posavsko hills	C5	46.573	16.202	12 Sep 2014	Yearling	18	17.37	2	0.580
LME177	Posavsko hills	C5	46.563	16.306	24 Sep 2014	Yearling	15	14.55	2	0.227
LME217	Posavsko hills	C5	46.408	15.340	13 Dec 2015	Yearling	16.5	17.26	2	0.505
LME221	Posavsko hills	C5	46.480	15.262	6 Sep 2015	Yearling	15	14.28	1	0.345
LME222	Posavsko hills	C5	46.563	16.280	7 Sep 2015	Yearling	9.5	8.79	0	0.246
LME225	Posavsko hills	C5	46.546	16.240	11 Sep 2015	Yearling	13.5	12.85	1	0.394
LME226	Posavsko hills	C5	46.573	16.202	3 Sep 2015	Yearling	13	14.23	2	0.362
LME227	Posavsko hills	C5	46.563	16.332	5 Sep 2015	Yearling	16.5	15.76	1	0.063
LME228	Posavsko hills	C5	46.635	16.321	5 Sep 2015	Yearling	14.5	13.76	1	0.421
LME291	Posavsko hills	C5	46.335	15.508	6 Dec 2015	Yearling	11.5	12.15	2	0.188

LME292	Posavsko hills	C5	46.299	15.534	19 Dec 2015	Yearling	11.5	12.35	1	0.453
LME293	Posavsko hills	C5	46.237	15.391	19 Dec 2015	Yearling	13.5	14.50	1	0.295
LME294	Posavsko hills	C5	46.255	15.352	19 Dec 2015	Yearling	11	12.35	1	0.368
LME296	Posavsko hills	C5	46.319	15.054	13 Dec 2014	Yearling	16	16.76	1	0.295
LME327	Posavsko hills	C5	46.668	15.668	26 Sep 2015	Yearling	16.5	16.08	1	0.543
LME328	Posavsko hills	C5	46.622	15.707	2 Oct 2015	Yearling	12.5	12.17	1	0.146
LME111	Pohorje	N1	46.182	14.445	7 Dec 2013	Yearling	17	17.67	2	0.201
**Sample ID**	**Area**	**Population**	**Latitude**	**Longitude**	**Date of harvest**	**Age class**	Body mass at harvest (kg)	**Standardized body mass (kg)**	**Number of corpora lutea (CL)**	**HL value**
LME115	Pohorje	N1	46.225	14.133	29 Oct 2014	Yearling	13	13.08	1	0.314
LME116	Pohorje	N1	46.180	14.121	28 Oct 2014	Yearling	16	16.06	2	0.223
LME117	Pohorje	N1	46.408	14.651	18 Sep 2014	Yearling	16	15.46	2	0.156
LME181	Pohorje	N1	46.381	15.418	5 Sep 2014	Yearling	15	14.26	2	0.231
LME182	Pohorje	N1	46.354	15.469	16 Oct 2014	Yearling	15	14.88	2	0.313
LME238	Pohorje	N1	45.984	15.660	20 Sep 2015	Yearling	13	12.49	1	0.314
LME239	Pohorje	N1	45.921	15.582	2 Oct 2015	Yearling	13	12.67	1	0.259
LME255	Pohorje	N1	45.852	13.714	10 Sep 2015	Yearling	11.5	10.84	1	0.314
LME256	Pohorje	N1	45.806	13.612	9 Sep 2015	Yearling	12.5	11.82	1	0.118
LME257	Pohorje	N1	45.815	13.599	22 Sep 2015	Yearling	11.5	11.02	1	0.295
LME258	Pohorje	N1	45.826	13.753	21 Oct 2015	Yearling	16.5	16.46	2	0.277
LME277	Pohorje	N1	46.021	14.718	6 Sep 2015	Yearling	9.5	8.78	2	0.294
LME278	Pohorje	N1	46.157	14.885	11 Sep 2015	Yearling	14	13.35	2	0.378
LME290	Pohorje	N1	45.547	13.785	6 Oct 2015	Yearling	12.5	12.23	1	0.309
LME77	Pohorje	N1	45.729	13.935	9 Oct 2013	Yearling	17	16.78	2	0.626
LME79	Pohorje	N1	46.466	16.095	14 Sep 2013	Yearling	14	13.40	1	0.251
LME83	Pohorje	N1	46.375	13.741	8 Sep 2013	Yearling	9.5	8.81	0	0.345
LME86	Pohorje	N1	46.458	13.947	6 Sep 2013	Yearling	15	14.28	2	0.363
LME87	Pohorje	N1	46.139	15.157	2 Oct 2013	Yearling	14.5	14.17	1	0.672
LME96	Pohorje	N1	46.039	14.705	13 Nov 2013	Yearling	10.5	10.81	0	0.423
LME120	Sub-Pannonian region	N2	45.984	15.634	21 Oct 2014	Yearling	17	16.96	2	0.249
LME141	Sub-Pannonian region	N2	46.002	15.621	9 Dec 2014	Yearling	16.5	17.20	1	0.460
LME183	Sub-Pannonian region	N2	45.756	13.922	4 Nov 2014	Yearling	11.5	11.67	2	0.267
LME184	Sub-Pannonian region	N2	45.746	13.896	10 Dec 2014	Yearling	15.5	16.21	2	0.129
LME203	Sub-Pannonian region	N2	45.737	13.897	30 Sep 2014	Yearling	16.5	16.14	2	0.237
LME205	Sub-Pannonian region	N2	45.665	13.885	30 Sep 2014	Yearling	18	17.64	2	0.352
LME241	Sub-Pannonian region	N2	45.592	13.758	24 Dec 2015	Yearling	12	12.93	2	0.395
LME243	Sub-Pannonian region	N2	45.993	15.621	6 Sep 2015	Yearling	14	13.28	2	0.500
**Sample ID**	**Area**	**Population**	**Latitude**	**Longitude**	**Date of harvest**	**Age class**	Body mass at harvest (kg)	**Standardized body mass (kg)**	**Number of corpora lutea (CL)**	**HL value**
LME244	Sub-Pannonian region	N2	45.984	15.634	7 Sep 2015	Yearling	15.5	14.79	2	0.508
LME245	Sub-Pannonian region	N2	45.853	13.727	12 Sep 2015	Yearling	15	14.37	2	0.295
LME246	Sub-Pannonian region	N2	45.746	13.896	13 Sep 2015	Yearling	15.5	14.88	1	0.378
LME247	Sub-Pannonian region	N2	46.085	13.631	27 Sep 2015	Yearling	15	14.60	1	0.202
LME248	Sub-Pannonian region	N2	45.719	13.884	3 Oct 2015	Yearling	15.5	15.17	2	0.345
LME249	Sub-Pannonian region	N2	45.537	13.696	8 Oct 2015	Yearling	14	13.76	2	0.444
LME250	Sub-Pannonian region	N2	45.879	13.700	20 Sep 2015	Yearling	16	15.49	2	0.334
LME251	Sub-Pannonian region	N2	45.888	13.649	12 Sep 2015	Yearling	13	12.37	1	0.259
LME259	Sub-Pannonian region	N2	46.063	14.071	21 Sep 2015	Yearling	16	15.50	2	0.312
LME260	Sub-Pannonian region	N2	46.351	14.105	19 Sep 2015	Yearling	18	17.47	2	0.515

LME261	Sub-Pannonian region	N2	46.597	14.976	4 Sep 2015	Yearling	15	14.25	2	0.256
LME262	Sub-Pannonian region	N2	46.597	14.963	10 Oct 2015	Yearling	15	14.79	1	0.271
LME263	Sub-Pannonian region	N2	46.498	15.432	26 Sep 2015	Yearling	13	12.58	0	0.362
LME264	Sub-Pannonian region	N2	46.764	16.023	24 Oct 2015	Yearling	16.5	16.50	2	0.277
LME265	Sub-Pannonian region	N2	46.011	15.647	22 Sep 2015	Yearling	14	13.52	1	0.431
LME270	Sub-Pannonian region	N2	45.755	13.896	2 Oct 2015	Yearling	17.5	17.17	1	0.424
LME271	Sub-Pannonian region	N2	45.756	13.922	26 Sep 2015	Yearling	17.5	17.08	2	0.314
LME112	Podravje and Slovenske gorice	N3	46.037	14.149	31 Dec 2013	Yearling	15.5	16.53	2	0.409
LME118	Podravje and Slovenske gorice	N3	46.137	14.484	27 Sep 2014	Yearling	15.5	15.10	2	0.248
LME130	Podravje and Slovenske gorice	N3	46.555	16.241	19 Dec 2014	Yearling	15.5	16.35	2	0.633
LME131	Podravje and Slovenske gorice	N3	46.635	16.334	6 Nov 2014	Yearling	14.5	16.70	1	0.690
LME133	Podravje and Slovenske gorice	N3	46.582	16.176	22 Oct 2014	Yearling	13.5	13.47	1	0.269
LME135	Podravje and Slovenske gorice	N3	46.354	14.716	22 Nov 2014	Yearling	18	18.44	2	0.328
LME210	Podravje and Slovenske gorice	N3	46.546	16.240	16 Dec 2014	Yearling	12	12.80	2	0.163
LME211	Podravje and Slovenske gorice	N3	46.328	15.002	30 Nov 2014	Yearling	12	12.56	2	0.414
LME212	Podravje and Slovenske gorice	N3	46.364	15.002	30 Nov 2014	Yearling	13.5	14.06	2	0.135
LME213	Podravje and Slovenske gorice	N3	46.364	15.002	11 Dec 2014	Yearling	15	15.73	0	0.202
LME298	Podravje and Slovenske gorice	N3	46.387	14.053	25 Oct 2015	Yearling	17	17.02	2	0.411
**Sample ID**	**Area**	**Population**	**Latitude**	**Longitude**	**Date of harvest**	**Age class**	Body mass at harvest (kg)	**Standardized body mass (kg)**	**Number of corpora lutea (CL)**	**HL value**
LME299	Podravje and Slovenske gorice	N3	46.281	14.444	20 Oct 2015	Yearling	16	15.94	1	0.309
LME300	Podravje and Slovenske gorice	N3	46.281	14.444	24 Oct 2015	Yearling	14.5	14.50	2	0.414
LME317	Podravje and Slovenske gorice	N3	46.300	15.313	27 Oct 2015	Yearling	16.5	16.55	1	0.385
LME84	Podravje and Slovenske gorice	N3	46.130	15.170	30 Oct 2013	Yearling	16	16.09	1	0.311
LME85	Podravje and Slovenske gorice	N3	46.130	15.157	1 Oct 2013	Yearling	12.5	12.16	1	0.231
LME121	Prekmurje	N4	46.054	14.084	25 Oct 2015	Yearling	17.5	17.52	1	0.269
LME138	Prekmurje	N4	46.081	14.148	11 Dec 2014	Yearling	13	13.73	2	0.121
LME151	Prekmurje	N4	45.635	14.912	7 Dec 2014	Yearling	15	15.67	2	0.462
LME152	Prekmurje	N4	45.644	15.104	27 Nov 2014	Yearling	16.5	17.02	2	0.480
LME153	Prekmurje	N4	45.599	15.091	6 Dec 2014	Yearling	16.5	17.15	2	0.461
LME154	Prekmurje	N4	45.554	14.797	7 Dec 2014	Yearling	15.5	16.17	2	0.276
LME155	Prekmurje	N4	45.842	15.182	9 Dec 2014	Yearling	20.5	21.20	2	0.378
LME156	Prekmurje	N4	45.841	15.233	7 Dec 2014	Yearling	15	15.67	2	0.499
LME281	Prekmurje	N4	45.720	13.948	17 Nov 2015	Yearling	18	18.37	2	0.295
LME282	Prekmurje	N4	45.520	13.734	1 Dec 2015	Yearling	14	14.58	1	0.598
LME283	Prekmurje	N4	45.738	13.948	2 Dec 2015	Yearling	16	16.59	2	0.409
LME284	Prekmurje	N4	45.738	13.948	8 Dec 2015	Yearling	14.5	15.18	1	0.284
LME285	Prekmurje	N4	45.764	13.896	18 Nov 2015	Yearling	15	15.38	2	0.522
LME286	Prekmurje	N4	46.503	16.018	15 Nov 2015	Yearling	17	17.34	2	0.331
LME287	Prekmurje	N4	46.384	16.250	7 Oct 2015	Yearling	15	14.75	2	0.445
LME288	Prekmurje	N4	46.466	16.121	17 Oct 2015	Yearling	15	14.90	2	0.303
LME289	Prekmurje	N4	46.503	16.005	22 Nov 2015	Yearling	16	16.44	2	0.408

Table S2. List of microsatellite loci used in the population genetic analysis of *Capreolus capreolus*.

PCR multiplex	Used primer pairs	Sequence 5 '– 3'	Fluorescent labelling	Fragment length	References
**SET1**	**BM1818**	AGCTGGGAATATAACCAAAGG<br>AGTGCTTTCAAGGTCCATGC	PET	247–263 bp	[124]
	**BM757**	TGGAAACAATGTAAACCTGGG<br>TTGAGCCACCAAGGAACC	NED	158–211 bp	[124]
	**CSSM66**	ACACAAATCCTTTCTGCCAGCTGA<br>AATTTAATGCACTGAGGAGCTTGG	FAM	167–185 bp	[125]
	**NVHRT73**	CTTGCCCATTTAGTGTTTTCT<br>TGCGTGTCATTGAATAGGAG	NED	210–267 bp	[126]
**SET2**	**NVHRT24**	TGTGGACTATAGGGAGC<br>GTGTACAAAAAGTGATTGAGT	FAM	97–125 bp	[126]
	**NVHRT48**	CGTGAATCTTAACCAGGTCT<br>GGTCAGCTTCATTTAGAAAC	PET	80–95 bp	[126]
	**NVHRT16**	ATTCTAAGCCCAAATAATCTT<br>TCTAAGGGGTCTGTGTCTT	NED	151–175 bp	[126]
	**RT1**	TGCCTTCTTTCATCCAACAA<br>CATCTTCCCATCCTCTTTAC	FAM	210–245 bp	[127]
**SET 3**	**MCM64**	TACAGTCCATGGGGTCACAAGAG<br>TCTGAATCTACTCCCTCCTCAGAGC	PET	123–153 bp	[125]
	**Roe1**	AAATTTGGCTCTGCAATCGG<br>ACACAAAAGCCACCCAATAC	PET	131–133 bp	[124]
	**ETH225**	GATCACCTTGCCACTATTTCCT<br>ACATGACAGCCAGCTGCTACT	NED	137–155 bp	[124]
	**Roe8**	AAGCCGCGCTTGAAGGAG<br>ATCAAGCTCCCCTCTTCG	FAM	59–101 bp	[124]
**SET 4**	**MAF70**	GCAGGACTCTACGGGCCTTGC<br>CACGGAGTCACAAAGAGTCAGACC	PET	117–155 bp	[124]
	**NVHRT21**	GCAGCGGAGAGGAACAAAAG<br>GGGGAGGAGCAGGGAAATC	PET	157–178 bp	[126]

Four multiplex PCRs for the amplification of microsatellite loci are presented.

Table S3. The null alleles frequency estimates by three different programmes.

Locus	FreeNA	Microchecker	Genepop
BM1818	0.013	0.041	0.012
BM757	0.014	0.009	0.004
CSSM66	0.018	0.024	0.018
NVHRT24	**0.058**	**0.070**	**0.056**
NVHRT48	0.018	0.037	0.036
NVHRT16	0.037	0.047	0.036
RT1	0.004	0.003	0.002
NVHRT21	0.015	0.049	0.012
Roe1	0.041	0.038	0.210
ETH225	**0.220**	**0.218**	**0.221**
Roe8	0.045	0.033	0.032
MCM64	0.005	0.015	0.012
MAF70	0.031	0.037	0.028

Null allele frequencies above 0.05 are indicated in bold. Two loci (ETH225 and NVHRT24) were excluded from the analyses.
